# Instrumental variable analysis in the context of dichotomous outcome and exposure with a numerical experiment in pharmacoepidemiology

**DOI:** 10.1186/s12874-018-0513-y

**Published:** 2018-06-22

**Authors:** Babagnidé François Koladjo, Sylvie Escolano, Pascale Tubert-Bitter

**Affiliations:** Biostatistics, Biomathematics, Pharmacoepidemiology and Infectious Diseases (B2PHI), Inserm, UVSQ, Institut Pasteur, Université Paris-Saclay, 16 Avenue Paul Vaillant-Couturier, Villejuif, 94807 France

**Keywords:** Instrumental variable, Nonlinear least squares, Logistic regression, Physician’s prescription preference, Pharmacoepidemiology, Observational studies, Simulation study

## Abstract

**Background:**

In pharmacoepidemiology, the prescription preference-based instrumental variables (IV) are often used with linear models to solve the endogeneity due to unobserved confounders even when the outcome and the endogenous treatment are dichotomous variables. Using this instrumental variable, we proceed by Monte-Carlo simulations to compare the IV-based generalized method of moment (IV-GMM) and the two-stage residual inclusion (2SRI) method in this context.

**Methods:**

We established the formula allowing us to compute the instrument’s strength and the confounding level in the context of logistic regression models. We then varied the instrument’s strength and the confounding level to cover a large range of scenarios in the simulation study. We also explore two prescription preference-based instruments.

**Results:**

We found that the 2SRI is less biased than the other methods and yields satisfactory confidence intervals. The proportion of previous patients of the same physician who were prescribed the treatment of interest displayed a good performance as a proxy of the physician’s preference instrument.

**Conclusions:**

This work shows that when analysing real data with dichotomous outcome and exposure, appropriate 2SRI estimation could be used in presence of unmeasured confounding.

## Background

In observational studies, unobserved confounding may biase the estimation of target effect. Over the last decade, this issue has recieved a growing attention in the field of epidemiological studies attempting to assess adverse effects of drugs with a few works focusing on instrumental variable (IV) approaches. Instrumental variable estimation is a well known approach for assessing endogeneity in statistical modelling [1, 2]. Endogeneity often arises when a causal model is poorly specified therby introducing a structural bias in the estimation of its parameters. This may result from a measurement error in variables [3], an unobserved variable [4] or an inverse causality between the outcome and some regressors. The general IV method of estimation attempts to remove this bias by using structural equations which incorporate instrumental variables in the model. Several theoretical approaches have been developed to build estimators of parameters and to study the properties of these estimators in causal models with endogeneity (see [5]). A well-known example is the case of linear models in which IV estimation leads to estimators with good properties of convergence such as consistency discussed in [6]. The structural bias can be completely removed in this case.

In pharmacoepidemiology, we most often deal with binary covariables (drug exposure), binary responses (adverse event indicator) and confounding variables which are variables that are correlated with exposure and response. A nonlinear model should be the first choice in this context to match with the specific nature of the variables. However, to quantify the risk of the adverse effect of a treatment in the presence of unobserved confounding, researchers investigating the IV-based estimation often model the probability of dichotomous events as a linear function of covariables, thus ignoring the basic features of a probability. Terza and colleagues [7] investigated the influences of mispecification on the estimation when a linear IV model is used in an inherently nonlinear regression setting with endogeneity. A substantial bias was demonstrated in their results. In the context of pharmacoepidemiology modelling, endogeneity is often due to unobserved confounding and various nonlinear IV methods such as the Generalized Method of Moment (GMM;[8, 9]) or the two-stage residual inclusion (2SRI; [10]) can be used to solve this issue. However in a review of IV methods, Klungel and colleagues [11] claimed that the GMM estimator with the logistic regression model is not consistent for causal Odd Ratio (OR) estimation owing to the non-collapsibility of the OR. Consistency is also not guaranteed for the 2SRI when the regression models are nonlinear in both stages. For these nonlinear IV methods, theoretical results exist under very restricted assumptions which do not cover the possible frameworks of real data. Overall, in the context of binary outcome several simulation studies investigate mainly 2-stage IV methods with the first step being linear and the second step being logistic as in [12]. A few articles concern double probit models (Chapman et al. [13]). Very few address the comparison of GMM and 2-stage approaches and none study GMM, 2-stage double logistic using the prescription preference-based instrumental variables.

In a simulation study, we compare the IV-based GMM and the 2SRI methods to the conventional method which does not account for endogeneity. These comparisons are based on the estimation of the regression coefficient of the exposure varible in nonlinear logistic model. Our numerical comparison of the methods involves several scenarios with different confounding levels and different instrument strengths for which computation formulas are established in the context of dichotomous outcome and exposure. We recall the general formula of the covariance matrix for the two-step estimation methods and give the corresponding expression for two-stage nonlinear least squares method in the context of logistic regressions.

The paper is organized as follows: we specify the model and describe the methods of estimation that will be analysed. Then we describe the simulation design, the criteria for evaluating the performances of the methods and the results of our simulations. The final sections discuss the results and make some concluding remarks. Details on the computation of the covariance matrix of the 2SRI method, the instrument’s strength and the confounding level are to be found in the appendices, as well as a detailed description of the simulation model and supplementary results.

## Methods

### Model

We consider a general model with dichotomous outcome and exposure that can be written as

1 $$  Y = F(\beta_{0} + T\beta_{t}+ X_{1}\beta_{1}+ X_{2}\beta_{2}+ X_{u}\beta_{u}) + e  $$

2 $$  \mathbb{E}(T|Z,X_{1},X_{2},X_{u}) = r(\alpha_{0} + Z\alpha_{z}+ X_{1}\alpha_{1}+ X_{2}\alpha_{2}+ X_{u})  $$

with *Y* and *T* as binary outcome (event or not) and treatment (*T*_1_ or *T*_2_) respectively, *X*_1_,*X*_2_ some covariables and *X*_*u*_ an unobserved confounder of the outcome and treatment. The function *F*(.)=*r*(.) denotes the logistic distribution function also known as *expit*(.): *expit*(.)=*exp*(.)/(1+*exp*(.)) and *e* denotes the error term. The parameter *β*=(*β*_0_,*β*_*t*_,*β*_1_,*β*_2_) denotes the vector of unknown parameters to be estimated. Without a confounding variable, all observed regressors are exogenous. In this case, the true model is written

3 $$  Y = F(\beta_{0} + T\beta_{t}+ X_{1}\beta_{1}+ X_{2}\beta_{2}) + e  $$

and conventional regression methods are suitable for estimating the parameters *β*. We will denote (3) *the conventional model*. If this model is adjusted to data in the presence of an unobserved confounder, the estimated coefficients would lead to a bias with a level depending on the confounding level. As the confounder *X*_*u*_ is not independent of treatment, the residuals of the conventional model are associated with the treatment. This causes endogeneity so a single regression of the outcome on observed covariables will fail to estimate *β*_*t*_ efficiently. A common strategy is to consider another regression model that links the endogenous variable with others. Equation (2) defines the auxiliary model that predicts treatment *T* as a function of covariables *X*_1_, *X*_2_, the confounder *X*_*u*_ and another variable *Z*. Variable *Z* denotes the instrumental variable (or instrument) related to the treatment, i.e. a variable correlated with the treatment and which has no direct association with the outcome.

The bias due to the unobserved confounder can significantly be reduced by means of the two-stage regression model using a valid instrument. As defined by Johnston and colleagues [14] and Greenland [15], a valid instrument must not be correlated with an unobserved confounder or with the error term in the true model (1). Formally, we assume that the instrument *Z* meets the following assumptions:

- ${\mathbb {C}ov}(Z,Y|T,X_{u},X_{1},X_{2})=0,$
- ${\mathbb {C}ov}(Z,T) \neq 0,$
- ${\mathbb {C}ov}(Z,X_{u}) = 0, {\mathbb {C}ov}(Z,X_{1}) = 0$ and ${\mathbb {C}ov}(Z,X_{2}) = 0$.

We also assume that the confounder *X*_*u*_ is not associated with the covariables *X*_1_ and *X*_2_, that is ${\mathbb {C}ov}(X_{u},X_{1}) = 0$ and ${\mathbb {C}ov}(X_{u},X_{2}) = 0$. The main goal is to estimate *β*_*t*_, the treatment coefficient which is the basis of risk evaluation; however an estimation of *β*_*t*_ is obtained in general by estimating vector *β* which is discussed below.

As already proved in the simple case of a linear model (for which the functions *F* and *r* are equal to identity in Eqs. (1) and (2)), a high association between the treatment and an instrument should improve the IV estimation of *β*. Finding a strong instrument is then a crucial step in all procedures of instrumental variable estimation.

In what follows, we first present some specific instrumental variables often used in pharmacoepidemiology, then discuss some IV estimators of *β* to obtain an estimate of *β*_*t*_ before addressing the properties of these estimators.

### Instrument in pharmacoepidemiology

An instrumental variable can be determined in many ways, provided that it meets the assumptions listed above. One of the problems is to find a valid instrument with a reasonable strength. The strength of an instrumental variable can be defined as resulting from the level of its association with the endogenous treatment. As such, it could be quantified by using the correlation coefficient between the treatment and the related instrument. In the wide range of pharmacoepidemiologic applications, we can summarize the various instrumental variables in three categories:

*Geographical variation.* Proximity to the care provider can positively influence access to treatment of a patient compared to others who live far away from health services. To account for this difference between patients, some researchers (see [16]) consider the distance between a patient and a care provider as an instrumental variable. Although this seems realistic as there is no direct association between this distance and the occurrence of disease, the presence or absence of health services can be associated with some socioeconomic characteristics. The latter are often considered as unmeasured confounders that call into question the suitability of using this instrument.

*Calendar time.* The use of calendar time as an instrument in pharmacoepidemiology often relies on the occurrence of an event that could change the attitude of the physician or patient regarding a treatment. This could be a change in guidelines for example or a change due to the arrival of a new drug on the market. The time from that event to the date of treatment defines the calendar time which clearly affects the outcome of the treatment since the change in physician or patient attitude will be more pronounced immediately after the event has occurred than later. An example of use of calendar time as an instrument can be found in [17].

*Physician’s prescription preference.* The most often used instrumental variables in pharmacoepidemiology are preference-based [18–20]. The issue is to compare the effectiveness of two treatments *T*_1_ and *T*_2_ when the assignment of treatment to the patient is not randomized. This is the case in observational studies where the prescriber of the treatment (the physician) introduces an effect that influences the outcome via the prescribed treatment. This effect results in the instrumental variable that reflects the influence of care-providers on the patient-treatment relationship. Brookhart and colleagues [21] define this instrumental variable as the “Physician’s Preference” (PP) and propose to use the treatment prescribed by a physician to its previous patient as a proxy of this IV for his/her new patient.

The instrumental variable $\left (Z_{i}^{*}\right)$ of the *i*^*th*^ patient will then be the treatment prescribed to the previous patient of the same physician. As a physician’s preference could change over time, Abrahamowicz and colleagues [22] introduce a new procedure to detect the change point and build a new proxy of PP that includes not only the treatment prescribed to the previous patient but all the previous prescriptions since the change point.

Those instrumental variables and some others are presented in a more detailed form in [23], [24, 25] or in [26] with enlightening discussion on their validity. In this work, we carry out a simulation study to examine how the proxy *Z*^∗^ of physician’s preference performs in the context of logistic regression. We also examine the physician’s preference-based IV in the continuous form (see [22]), i.e. the proportion (*pr*) of all previous patients of the same physician who were prescribed the treatment of interest. This corresponds to the empirical estimator of the probability for a physician to prefer the treatment of interest.

### IV Estimation of *β*_*t*_

Estimating *β*_*t*_ in model (1) with an unobserved confounder *X*_*u*_ amounts to estimating vector *β* and taking the corresponding component of treatment *T*. Below, we present two methods that can provide consistent estimation of *β* and then that of *β*_*t*_: the two-stage residual inclusion (2SRI) method and the generalized method of moment (GMM).

The two-stage residual inclusion method is a modified version of the two-stage least squares (2SLS) method used to estimate the parameters in linear models with instrumental variables. As mentioned by Greene [5], the first stage of the 2SLS method predicts the endogenous variable (the treatment here) using the instruments and other covariables (Eq. (2)). In the second stage, the endogenous variable is just replaced by its prediction from the first stage. This method is called two-stage predictor substitution (2SPS) when the first stage is nonlinear. Unlike the 2SLS, the residuals of the first regression serve as a regressor in the second stage of 2SRI method. This method also generalizes to the nonlinear models i.e. when first and second stages are nonlinear. The rationale of this approach can be intrinsically related to the form of the true model: sometimes, the prediction equation of the outcome includes the error term of the auxiliary regression. An example is the case when the confounder is the only source of error in the auxiliary regression as considered in [27]. In a linear model, both 2SPS and 2SRI approaches are equivalent.

The GMM is an alternative method for obtaining a reliable estimator of parameter *β* in a model with an endogenous variable. It is based on the classical assumption

4 $$  \mathbb{E}(e|T,X_{1},X_{2},X_{u}) =0  $$

of the error term in Eq. (1). This assumption does not hold in general because the confounder *X*_*u*_ is unobserved (i.e. $\mathbb {E}(e|T,X_{1},X_{2}) \neq 0)$. In this case, the treatment is endogenous and its coefficient *β*_*t*_ cannot be consistently estimated. One suppose in general that there exists some observable instruments *w*_1_ such that $\mathbb {E}(e|w) = 0$ with *w*=(*w*_1_,*X*_1_,*X*_2_). Typically, *w* corresponds to the vector of exogenous and endogenous variables with endogenous regressors replaced by their corresponding instruments. Using the law of iterated expectation, the last condition implies

5 $$  \mathbb{E}(e.w) = 0.  $$

The method of moment solves the empirical version of (5) in *β*, i.e. $\frac {1}{n} \sum _{i = 1}^{n} e_{i}w_{i} = 0$ where *n* is the sample size, *e*_*i*_ is the *i*^*th*^ component of *e* and *w*_*i*_ the *i*^*th*^ row of *w*. In turn, the GMM minimizes the quadratic form

6 $$  q(\beta) = \left(\frac{1}{n} \sum_{i = 1}^{n} e_{i}w_{i} \right)^{'}\Omega \left(\frac{1}{n} \sum_{i = 1}^{n} e_{i}w_{i}\right)  $$

where *Ω* denotes a weighting matrix. As discussed in [28], there are several choices for matrix *Ω* leading to different estimators of *β*. The optimal approach is to define *Ω* as the inverse of the asymptotic covariance matrix (depending on *β*) of the estimator. Some alternative procedures are also suggested by Hansen and colleagues [29].

### Properties

The properties of the 2SRI are addressed by Terza in [27] when the residual also acting as unobserved confounder in the first-stage regression (at Eq. (2)) is additive. Under this assumption and using nonlinear least squares regression in each stage, they show the consistency of the estimator $\hat {\beta }$ from the second stage. Since the confounder is not additive in the model (2), the first stage estimate of a 2SRI will not be consistent, nor will $\hat {\beta }$. The residual from the first-stage is indeed an unknown function of an unobserved confounder. Then there is a bias that depends on the form of this unknown function when one applies the 2SRI method to the structural model at Eqs. (1) and (2). The derivation of the covariance matrix of 2SRI estimator follows a two-step regression covariance matrix of the form

7 $${} \begin{aligned} {\mathbb{V}ar}(\hat{\beta}) &=\! \left(\!A_{22}^{-1} S_{2} A_{22}^{-1^{\prime}}\!\right)\!\!/n +\! \left(\!A_{22}^{-1}A_{21} A_{11}^{-1} S_{1}A_{11}^{-1^{\prime}}A_{21}^{\prime} A_{22}^{-1^{\prime}}\!\right)\!\!/n\\ &\quad- \left(A_{22}^{-1} S_{21} A_{11}^{-1^{\prime}}A_{21}^{\prime} A_{22}^{-1^{\prime}}\right)/n, \end{aligned}  $$

where the computation of matrices *A* and *S* is given in Appendix A.

The GMM is a well documented estimation procedure. Both in linear and nonlinear models, several results on the estimator have already been established. In the literature on econometric analysis, the nonlinear GMM with an instrumental variable has received particular attention. In the pioneering work by Amemiya [30], the author demonstrated the consistency and derived the asymptotic distribution of the nonlinear two-stage least squares estimator (NL2SLS).

This result provided an important insight into how to handle nonlinear models with endogeneity. Later, Hansen [31] showed the asymptotic properties (consistency and asymptotic distribution) of the GMM to be a kind of generalization of the NL2SLS. More recently, Cameron and Trivedi [28] reviewed the method and gave details on the computation of the estimator’s covariance matrix in some specific cases. Despite the different results on the GMM with an instrumental variable, its performances in terms of bias and variance depend on the validity and strength of the instrument and the nature of the variables in the model. The computation of the covariance matrix of GMM is given in [28] and implemented in dedicated softwares of which [32] is a good example.

Below, we investigate the performances of these methods with numerical experiments.

### Simulation design and data generation

A numerical experiment was conducted to investigate and compare several methods of IV estimation in the context of a dichotomous outcome and exposure with endogeneity in pharmacoepidemiology. In this experiment, we cover a wide range of possible scenarios. We choose values of parameter *α*=(*α*_0_,*α*_*z*_,*α*_1_,*α*_2_) corresponding to some values of correlation between the variable *T*^∗^=*α*_0_+*PPα*_*z*_+*X*_1_*α*_1_+*X*_2_*α*_2_+*X*_*u*_ and the Physician’s prescribing Preference instrument *PP*. In fact, we keep *α*_0_, *α*_1_ and *α*_2_ fixed and only *α*_*z*_ varies from a scenario to the other. The computation of this correlation is given in Appendix B. It somewhat reflects the strength of the instrument when the confounder and other covariables are kept fixed. For each value of the instrument’s strength, there are three levels of confounding measured by the standard deviation *σ*_*u*_ of the confounding variable *X*_*u*_, *σ*_*u*_∈{0.5,1,1.5} which leads to a set of correlations between *T*^∗^ and *X*_*u*_. We then have nine scenarios of strengths and confounding level.

For each scenario, we generate *ns*=1000 Monte Carlo samples of size *n*, *n*=10000,20000 and 30000. The number of patients per physician is kept fixed and equals 100; the confounder *X*_*u*_ and covariates *X*_1_ and *X*_2_ are assumed to have the normal distributions *N*(0,*σ*_*u*_), *N*(−2,1) and *N*(−3,1) respectively and the physician’s prescribing preference has the Bernoulli distribution *B*(0.7). We first simulate the covariables *X*_1_ and *X*_2_, the confounder *X*_*u*_ and the physician’s prescribing preference which is the same for all the patients of the same physician. Using the already fixed values of parameters *α*, the probability *p*_*i*_ that patient *i* will be prescribed the drug of interest is calculated by inverting the logit function, i.e. *p*_*i*_=*F*(*α*_0_+*PP*_*i*_*α*_*z*_+*X*_1*i*_*α*_1_+*X*_2*i*_*α*_2_+*X*_*ui*_). Treatment *T*_*i*_ of patient *i* is then generated as a Bernoulli realisation with parameter *p*_*i*_. The same procedure as for the treatment is used to simulate for each patient *i*, the corresponding outcome *y*_*i*_. We fixed the parameter *α* such that the proportion of exposed patients ranges between 2 and 6% and the prevalence of the event of interest is chosen to be smaller than 5% to reflect a real-life situation of a new treatment (not frequently prescribed) and rare adverse event. With the fixed value of *β*, we compute the probability *F*(*β*_0_+*T*_*i*_*β*_*t*_+*X*_1*i*_*β*_1_+*X*_2*i*_*β*_2_+*X*_*ui*_*β*_*u*_) of the event for patient *i* and then simulate his outcome *y*_*i*_. We also explored a more balanced situation in term of exposure frenquencies (between 26 and 45*%*).

Finally, proxy *Z*^∗^ of PP is the treatment given to the previous patient and the continuous instrument *pr* is the proportion of patients of the same physician who were previously prescribed the treatment of interest. More details on the simulation model and the data generating R code are given in Appendix D.

### Estimation methods

For the true and conventional models which do not assume endogeneity, the classical one-step regression method without instrumental variable is used. The estimations are performed with the existing regression functions (glm and nls) implemented in R statistical software (R Development core team 2008) in the R package stats.

For the 2SRI method, a two-step regression is used following the procedure outlined in the section dedicated to IV estimation procedure. Recall that the covariance matrices of $\hat {\beta }$ from the second step regression for both methods retrieved from the software results will not be valid since their calculation ignores the fact that some estimated parameters from the first-stage regression are included in the second stage. Then, we re-evaluated these covariance matrices using the sequential two-step estimation procedure taking into account the fact that an estimated variable is used in the second step. The computation of these covariance matrices is given in Appendix A.

For the GMM, the R package gmm proposed by Chaussé [32] is a very helpful tool for computating parameters and estimating covariance. The user needs to implement the sample version of the moment condition function at Eq. (5) and its gradient if possible; if not, a numerical approximation of the gradient function will be used by the gmm function to perform the estimation.

From these estimations, we calculate the asymptotic covariance matrices and the corresponding confidence intervals whose levels are evaluated below.

### Evaluation criteria

To evaluate the performances of the various methods, we consider several criteria including the percentage of relative bias (rB in *%*) defined as

$$\text{rB} = 100*\frac{1}{ns}\sum_{j=1}^{ns}\left(\frac{\hat{\beta}_{t}^{(j)}}{\beta_{t}}-1\right);$$

the asymptotic standard deviation estimated by the square root of the Monte-Carlo mean of variance $\bar {\hat {\sigma }}^{2} = \frac {1}{ns}\sum _{j=1}^{ns} \hat {\sigma }_{j}^{2},$ with $\hat {\sigma }_{j}^{2}$ the asymptotic variance of $\hat {\beta }_{t}^{(j)}$ and the Monte-Carlo estimator of the true variance ${\mathbb {V}ar}(\hat {\beta }_{t}) = \frac {1}{ns-1}\sum _{j=1}^{ns} \left (\hat {\beta }_{t}^{(j)}- \bar {\hat {\beta }_{t}}\right)^{2}$. We also consider the square root of the mean squares error rMSE given by

$$\text{rMSE} = \sqrt{\frac{1}{ns}\sum_{j=1}^{ns}\left(\hat{\beta}_{t}^{(j)}-\beta_{t} \right)^{2}},$$

and the lower and upper non-coverage probabilities (in *%*) defined as

$${Er}_{inf} = 100*\frac{1}{ns}\sum_{j=1}^{ns}1_{\left[ \beta_{t} < {IC}_{inf}^{(j)}\right]}$$

and

$${Er}_{sup} = 100*\frac{1}{ns}\sum_{j=1}^{ns}1_{\left[ \beta_{t} > {IC}_{sup}^{(j)}\right]} $$

where $ IC^{(j)} = \left [{IC}_{inf}^{(j)};{IC}_{sup}^{(j)}\right ]$ denotes the confidence interval of *β*_*t*_ from the *j*^*th*^ Monte-Carlo sample using the asymptotic distribution of $\hat {\beta }_{t}^{(j)}$. The nonparametric bootstrap based estimate of variance and non-coverage probabilities are also investigated in these simulations and the results are analysed below. We complete all these criteria by the equivalent of the first-stage F-statistics in linear regression (see [33]) testing instrument exclusion in the treatment choice model.

## Results

**Fig. 1:**
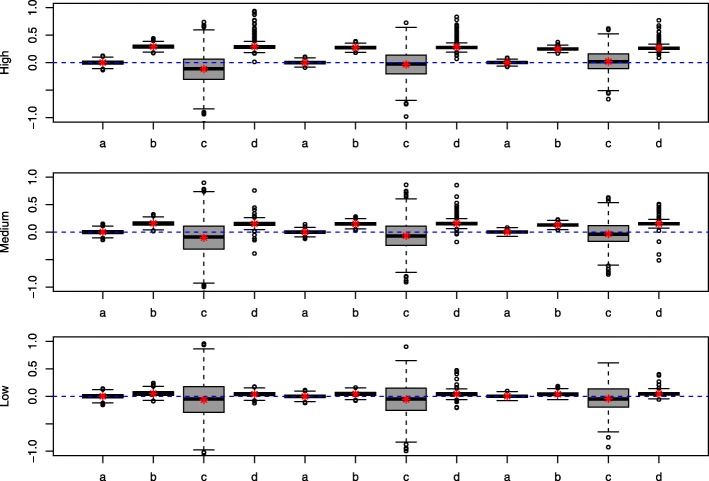
Relative bias (rB) of the methods. a: True model b: conventional model; c : 2SRI with instrument pr; d: GMM with instrument pr. Low, Medium and High indicate the corresponding level of confounding and the instrument strength grows from a, b, c, d sequence to the next (from left to right)

Table 1 summarizes the performances of each method in terms of relative bias (rB), the standard deviation (sd), the square root of mean squares error (rMSE) and the non-coverage probabilities (pval=*Er*_*inf*_+*Er*_*sup*_). It presents the results related to instrument *pr*. There were only slight differences between the results with instrument *pr* and those with *Z*^∗^, so we omitted the results related to instrument *Z*^∗^. For some samples, the GMM fails to converge owing to singularity problems in the covariance matrix. The estimations from these samples are simply removed (cases marked ‘ −’ in Table 1) for all methods. For the other scenarios, infinite variance estimate or outlier coefficient estimate may be observed; the corresponding samples were dropped for the calculation of the criteria. Table 2 shows the number of samples leading to an outlier estimation (rB>100*%* or infinite variance) among the 1000 simulated samples. Figure 1 complements the results in Table 1 by displaying the boxplot distributions of rB. Each series of letters a,b,c and d corresponds to the results related to an instrument strength with each letter corresponding to a method as detailed in the legend of Fig. 1. In Table 1 the sd values refer to the Monte-Carlo-based standard deviation. Except for the GMM estimator where outlier values for the asymptotic variance were observed, the Monte-Carlo-based standard deviation, the bootstrap-based estimate (not shown) and the asymptotic estimate were very close.

**Table 1:** Performances of methods using instrument *pr*

		Instrument strength											
		Weak				Mod				Strong			
Level	Method	rB	sd	rMSE	pval	rB	sd	rMSE	pval	rB	sd	rMSE	pval
30000													
High	Tr	0.19	0.12	0.12	0.05	0.21	0.10	0.10	0.06	0.14	0.08	0.08	0.04
	Conv	29.28	0.12	0.89	1.00	27.53	0.10	0.83	1.00	25.10	0.08	0.76	1.00
	2SRI	− 11.44	0.86	0.92	0.06	− 3.44	0.73	0.74	0.04	1.92	0.58	0.58	0.04
	GMM	29.37	0.23	0.91	0.38	28.39	0.19	0.87	0.77	26.69	0.13	0.81	0.93
Med	Tr	0.23	0.13	0.13	0.06	0.12	0.10	0.10	0.05	0.19	0.09	0.09	0.05
	Conv	15.95	0.13	0.50	1.00	14.90	0.11	0.46	1.00	13.10	0.10	0.40	1.00
	2SRI	− 9.75	0.97	1.01	0.07	− 6.35	0.80	0.82	0.05	− 3.40	0.66	0.66	0.05
	GMM	15.35	0.18	0.49	0.29	15.72	0.17	0.50	0.56	15.28	0.15	0.48	0.89
Low	Tr	0.10	0.14	0.14	0.06	− 0.13	0.11	0.11	0.04	0.18	0.10	0.10	0.04
	Conv	4.92	0.15	0.21	0.72	4.39	0.12	0.18	0.67	3.95	0.12	0.17	0.56
	2SRI	− 6.32	1.04	1.06	0.06	− 5.49	0.86	0.87	0.04	− 4.23	0.73	0.75	0.04
	GMM	3.80	0.14	0.18	0.30	4.15	0.14	0.19	0.39	4.61	0.12	0.18	0.84
20000													
High	Tr	0.27	0.14	0.14	0.04	0.05	0.12	0.12	0.05	0.30	0.10	0.10	0.06
	Conv	29.53	0.14	0.90	1.00	27.51	0.11	0.83	1.00	25.21	0.11	0.76	1.00
	2SRI	− 11.03	1.05	1.10	0.05	−5.51	0.91	0.92	0.04	1.34	0.70	0.70	0.04
	GMM	29.01	0.22	0.90	0.32	28.03	0.17	0.86	0.72	27.28	0.19	0.84	0.92
Med	Tr	0.09	0.16	0.16	0.06	0.22	0.13	0.13	0.06	0.19	0.11	0.11	0.07
	Conv	15.94	0.16	0.50	0.99	15.06	0.14	0.47	1.00	13.31	0.13	0.42	0.99
	2SRI	− 10.08	1.19	1.23	0.06	−6.69	1.00	1.02	0.05	− 3.67	0.79	0.80	0.04
	GMM	15.01	0.18	0.48	0.25	15.64	0.19	0.50	0.53	15.40	0.17	0.49	0.85
Low	Tr	− 0.24	0.12	0.12	0.06	0.11	0.14	0.14	0.06	−	−	−	−
	Conv	3.58	0.15	0.19	0.42	4.71	0.16	0.21	0.61	−	−	−	−
	2SRI	0.04	0.88	0.89	0.04	−5.92	1.09	1.10	0.04	−	−	−	−
	GMM	4.41	0.16	0.22	0.79	4.15	0.16	0.20	0.39	−	−	−	−
10000													
High	Tr	0.03	0.21	0.21	0.05	0.49	0.16	0.16	0.05	0.43	0.14	0.14	0.06
	Conv	29.69	0.20	0.91	1.00	28.22	0.16	0.86	1.00	25.70	0.15	0.79	1.00
	2SRI	− 14.47	1.64	1.70	0.07	−2.71	1.27	1.27	0.04	1.19	1.04	1.04	0.05
	GMM	28.86	0.25	0.90	0.30	28.61	0.24	0.89	0.68	27.45	0.22	0.85	0.90
Med	Tr	−	−	−	−	0.58	0.18	0.18	0.04	0.48	0.15	0.16	0.05
	Conv	−	−	−	−	15.83	0.19	0.51	0.97	13.78	0.17	0.45	0.93
	2SRI	−	−	−	−	−7.42	1.41	1.43	0.05	−3.57	1.13	1.13	0.03
	GMM	−	−	−	−	15.74	0.23	0.52	0.50	15.66	0.21	0.51	0.80
Low	Tr	−	−	−	−	0.08	0.20	0.20	0.05	−0.36	0.17	0.17	0.07
	Conv	−	−	−	−	5.27	0.22	0.27	0.55	3.88	0.20	0.23	0.37
	2SRI	−	−	−	−	−6.12	1.61	1.62	0.05	−4.37	1.31	1.31	0.04
	GMM	−	−	−	−	3.77	0.24	0.27	0.36	3.85	0.21	0.24	0.74

Legend: Tr = True model, Conv = Conventional model, 2SRI = Two-Stage Residual Inclusion, GMM = Generalized Method of Moment. Low, Med (Medium), High denote the level of confounding whereas Weak, Mod (Moderate), Strong stand for instrument strength. For the criteria, rB = relative bias (*%*), sd = standard deviation, rMSE = root Mean Squares Error and pval = non-coverage probabilities. The numbers 10000, 20000 and 30000 stand for different sample sizes

**Table 2:** Number of samples among 1000 leading to outliers in GMM estimation

*n*	Level	Weak	Mod	Strong
30000				
	High	155	61	31
	Med	78	65	43
	Low	41	149	65
20000				
	High	149	70	64
	Med	64	72	43
	Low	25	124	−
10000				
	High	95	58	37
	Med	−	78	55
	Low	−	110	44

Legend: Low, Med (Medium), High denote level of confounding whereas Weak, Mod (Moderate), Strong stand for instrument strength. The number *n* with values 10000, 20000 and 30000 stands for the sample size

As expected, the relative bias shows that the estimation from the true and conventional models are insensitive to instrument strength but the confounding level affects the estimation in the conventional model: the relative bias increases with level of confounding. In the presence of a strong instrument, the 2SRI tends to improve the estimate when the level of confounding increases. This trend is reversed when the instrument is weak, i.e. the relative bias and the confounding level have the same direction of variation. The percentage of relative bias of the GMM does not seem too sensitive to instrument strength: it just changes slightly when the strength of the instrument grows. However, this bias increases with the magnitude of confounding, which shows the impact of endogeneity on this method (See Fig. 1).

For the standard deviation (sd) and the square root of the mean squares error (rMSE), the asymptotic results (*n*=30000) show that both criteria decrease when the level of confounding or instrument strength grows, this being the case for all methods except the GMM for which the rMSE decreases very slowly or remains almost constant in some cases. This trend confirms the already obverved low sensitivity of the GMM to the strength of the instruments used in this simulation. Even though the 2SRI has the larger sd than the other methods in all scenarios with an impact on rMSE in several cases, rMSE for 2SRI method seems improved with high confounding and a strong instrument.

Concerning the non coverage probabilities (pval), the true model estimation and the 2SRI displayed an estimated non-coverage probability around the nominal level of 5% in almost all scenarios. Their values ranged from 4 to 6% and reached 7% in rare cases. The non-coverage probability was very large for the other methods, even with large samples: the results showed an overestimation of the coefficient of treatment for the GMM and conventional approaches. Even at low confounding levels, the conventional method yields very poor coverage probabilities which is coherent with what was observed for the relative bias.

We observe that the metric of the instrument we use retains the same direction of variation with the F-statistics eequivalents (Tables 3 and 4 of Appendix C). Finally, Table 5 in Appendix D summarizes the performances of each method for more balanced exposure frequencies and large sample size (*n*=30000). In general, performances are less good in comparison with small exposure frequency situation. One could also note that numerical problems arise more often (see Table 6 of Appendix D). Nevertheless, 2SRI is better in term of relative bias and non coverage probability than the conventional method and GMM.

Overall, the results are satisfactory for the 2SRI approach which achieves a similar level of performance to the true model regarding estimation of the confidence interval in the imbalanced situation. We close this section by pointing out the strong numerical instability observed when computing the GMM estimator during these simulations. This could explain the modest performance displayed by the GMM in the simulation results.

## Discussion

In this paper, we focus on the effectiveness of regression coefficient estimation in a context of endogeneity, particularly the endogeneity due to unobserved confounding. We are interested in the coefficient of an endogenous treatment which is the basis of risk assessment in pharmacoepidemiology. Linear models are often used in this context to model the probability of dichotomous events (see [7]). Through a simulation study, we investigate the behavior of parameter estimation in nonlinear models specifically logistic regression using some IV-based methods that could potentially be used to overcome the endogeneity issue. The simulation study also made it possible to assess two preference-based instrumental variables in pharmacoepidemiology.

The results reported from the simulation study show that the 2SRI using nonlinear regression at each stage is an interesting alternative for estimating the coefficient of the endogenous treatment in a logistic regression model. It is very simple to implement and yields satisfactory results regarding the bias and the confidence interval estimate. It was found to yield the most accurate estimate of non-coverage probabilities and thus a more accurate estimate of confidence intervals among the IV-based methods that were compared. However, the conventional approach behaved better than the 2SRI in some cases, especially when the confounding level was weak. We believe that in these cases, the level of confounding is not sufficiently high to require the use of an instrument in the estimation. However, to our knowledge there is still no way to assess the level of unmeasured confounding. For the GMM, the estimation procedure was remarkably unstable. That instability may be attributable to the dichotomous nature of the variables (outcome and exposure) in the context of pharmacoepidemiology with the preference-based instrument. This situation makes the GMM approach is not to be recommended in this context unless another instrument has proved to behave satisfactorily with this method.

Concerning the instruments under investigation in this study, the proportion of all previous patients of the same physician who were prescribed the treatment of interest proved to be a good proxy of the physician’s preference instrument. This instrument was previously considered by Abrahamowicz and colleagues [22] for investigating the detection of a possible change point in the physician’s preference and their results also seemed satisfactory. This proxy of the physician’s preference instrument is thus a credible alternative to the well known other proxy based only on the single patient of the same physician who was prescribed the treatment of interest.

Even though this work throws light on the performances of IV estimators in the context of a nonlinear model with endogeneity, more work is needed to explore the behavior of these estimators in other contexts when the prevalence of exposure and /or outcome varies.

## Conclusions

In observational studies, when assessing the effect of drug exposure on a dichotomous outcome, investigators could use appropriate 2SRI estimation to account for unmeasured confounding. This work showed that two logistic regressions as well as a physician’s preference proxy for IV yeald satisfactory results.

## Appendix A: Asymptotic variance of the nonlinear 2SRI

Using the sequential two-step estimation procedure, the nonlinear 2SRI estimator minimizes the least squares criterion

8 $$ Q(\beta) = \frac{1}{2n}\sum_{i=1}^{n} \left(y_{i}-F_{\hat{\alpha}}\left(X_{i\hat{\alpha}}^{\prime}\beta\right)\right)^{2}  $$

where $\hat {\alpha }$ denotes the nonlinear least squares estimator of *α* from the first stage. If we set $\eta _{i\beta } = X_{i\hat {\alpha }}^{\prime }\beta,$ the first-order condition in *β* is given by

9 $$  - \frac{1}{n}\sum_{i=1}^{n} \frac{\partial F_{\hat{\alpha}}}{\partial \eta_{i\beta}}(\eta_{i\beta})\frac{\partial \eta_{i\beta}}{\partial{\beta} } \left(y_{i}-F_{\hat{\alpha}}(\eta_{i\beta})\right) = 0.  $$

Given that $\hat {\alpha }$ is a consistent estimator of *α*_0_, the Taylor-lagrange expansion of (9) arround the true value (*α*_0_,*β*_0_) gives

$$\begin{aligned} 0 = \frac{1}{n}\sum_{i=1}^{n} \frac{\partial F_{\alpha}}{\partial \eta_{i\beta}}(\eta_{i\beta})\frac{\partial \eta_{i\beta}}{\partial{\beta}} \left(y_{i}-F_{\alpha}(\eta_{i\beta})\right)_{(\alpha_{c};\beta_{c})} +\\ \left(\frac{1}{n}\sum_{i=1}^{n} \frac{\partial^{2} F_{\alpha}}{\partial \eta_{i\beta} \partial \eta_{i\beta}^{'} }(\eta_{i\beta})\frac{\partial \eta_{i\beta}}{\partial{\alpha}} \frac{\partial \eta_{i\beta}}{\partial{\beta^{\prime}}} \left(y_{i}-F_{\alpha}(\eta_{i\beta})\right) \right)_{(\alpha_{c};\beta_{c})} (\hat{\alpha}-\alpha_{0}) +\\ \left(\frac{1}{n}\sum_{i=1}^{n} \frac{\partial F_{\alpha}}{\partial \eta_{i\beta}}(\eta_{i\beta})\frac{\partial^{2} \eta_{i\beta}}{\partial{\alpha} \partial{\beta^{\prime}} } \left(y_{i}-F_{\alpha}(\eta_{i\beta})\right) - \frac{1}{n}\sum_{i=1}^{n} \frac{\partial F_{\alpha}}{\partial \eta_{i\beta}}(\eta_{i\beta})\frac{\partial \eta_{i\beta}}{\partial{\alpha} } \frac{\partial \eta_{i\beta}}{\partial{\beta{\prime}}}\frac{\partial F_{\alpha}}{\partial \eta_{i\beta}}(\eta_{i\beta}) \right)_{(\alpha_{c};\beta_{c})} (\hat{\alpha}-\alpha_{0}) +\\ \left(\frac{1}{n}\sum_{i=1}^{n} \frac{\partial^{2} F_{\alpha}}{\partial \eta_{i\beta} \partial \eta_{i\beta}^{\prime} }(\eta_{i\beta})\frac{\partial \eta_{i\beta}}{\partial{\beta}} \frac{\partial \eta_{i\beta}}{\partial{\beta^{\prime}}} \left(y_{i}-F_{\alpha}(\eta_{i\beta})\right) - \frac{1}{n}\sum_{i=1}^{n} \frac{\partial F_{\alpha}}{\partial \eta_{i\beta}}(\eta_{i\beta})\frac{\partial \eta_{i\beta}}{\partial{\beta} } \frac{\partial \eta_{i\beta}}{\partial{\beta{\prime}}}\frac{\partial F_{\alpha}}{\partial \eta_{i\beta}}(\eta_{i\beta}) \right)_{(\alpha_{c};\beta_{c})} (\hat{\beta}-\beta_{0}) \end{aligned} $$

for some (*α*_*c*_;*β*_*c*_) between $(\hat {\alpha };\hat {\beta })$ and (*α*_0_;*β*_0_). Under the assumption ${\mathbb {E}}\left (\frac {\partial {Q}}{\partial {\beta }}\right)_{\beta _0} = 0$ the terms involving the residuals $\left (y_{i}-F_{\alpha }(\eta _{i\beta })\right)$ in the above expansion, except the first, all tend in probability to zero and we obtain

$$\begin{aligned} \sqrt{n}(\hat{\beta}-\beta_{0}) \approx \left(\frac{1}{n}\sum_{i=1}^{n} \frac{\partial F_{\alpha}}{\partial \eta_{i\beta}}(\eta_{i\beta})\frac{\partial \eta_{i\beta}}{\partial{\beta} } \frac{\partial \eta_{i\beta}}{\partial{\beta^{\prime}}}\frac{\partial F_{\alpha}}{\partial \eta_{i\beta}}(\eta_{i\beta}) \right)_{(\alpha_{0};\beta_{0})}^{-1} \left(\frac{1}{\sqrt{n}}\sum_{i=1}^{n} \frac{\partial F_{\alpha}}{\partial \eta_{i\beta}}(\eta_{i\beta})\frac{\partial \eta_{i\beta}}{\partial{\beta}} \left(y_{i}-F_{\alpha}(\eta_{i\beta})\right)\right)_{(\alpha_{0};\beta_{0})}\\ - \left(\frac{1}{n}\sum_{i=1}^{n} \frac{\partial F_{\alpha}}{\partial \eta_{i\beta}}(\eta_{i\beta})\frac{\partial \eta_{i\beta}}{\partial{\beta} } \frac{\partial \eta_{i\beta}}{\partial{\beta^{\prime}}}\frac{\partial F_{\alpha}}{\partial \eta_{i\beta}}(\eta_{i\beta}) \right)_{(\alpha_{0};\beta_{0})}^{-1} \left(\frac{1}{n}\sum_{i=1}^{n} \frac{\partial F_{\alpha}}{\partial \eta_{i\beta}}(\eta_{i\beta})\frac{\partial \eta_{i\beta}}{\partial{\alpha} } \frac{\partial \eta_{i\beta}}{\partial{\beta^{\prime}}}\frac{\partial F_{\alpha}}{\partial \eta_{i\beta}}(\eta_{i\beta}) \right)_{(\alpha_{0};\beta_{0})} (\hat{\alpha}-\alpha_{0}). \end{aligned} $$

The quantity $\sqrt {n}(\hat {\beta }-\beta _{0})$ may then be written

$$\begin{aligned} \sqrt{n}(\hat{\beta}-\beta_{0}) &\approx \left(\frac{1}{n}\sum_{i=1}^{n} \frac{\partial F_{\alpha}}{\partial \eta_{i\beta}}(\eta_{i\beta})\frac{\partial \eta_{i\beta}}{\partial{\beta} } \frac{\partial \eta_{i\beta}}{\partial{\beta^{\prime}}}\frac{\partial F_{\alpha}}{\partial \eta_{i\beta}}(\eta_{i\beta}) \right)_{(\alpha_{0};\beta_{0})}^{-1} \left(\frac{1}{\sqrt{n}}\sum_{i=1}^{n} \frac{\partial F_{\alpha}}{\partial \eta_{i\beta}}(\eta_{i\beta})\frac{\partial \eta_{i\beta}}{\partial{\beta}} \left(y_{i}-F_{\alpha}(\eta_{i\beta})\right)\right)_{(\alpha_{0};\beta_{0})}\\ &\quad- \left(\frac{1}{n}\sum_{i=1}^{n} \frac{\partial F_{\alpha}}{\partial \eta_{i\beta}}(\eta_{i\beta})\frac{\partial \eta_{i\beta}}{\partial{\beta} } \frac{\partial \eta_{i\beta}}{\partial{\beta^{\prime}}}\frac{\partial F_{\alpha}}{\partial \eta_{i\beta}}(\eta_{i\beta}) \right)_{(\alpha_{0};\beta_{0})}^{-1} \left(\frac{1}{n}\sum_{i=1}^{n} \frac{\partial F_{\alpha}}{\partial \eta_{i\beta}}(\eta_{i\beta})\frac{\partial \eta_{i\beta}}{\partial{\alpha} } \frac{\partial \eta_{i\beta}}{\partial{\beta^{\prime}}}\frac{\partial F_{\alpha}}{\partial \eta_{i\beta}}(\eta_{i\beta}) \right)_{(\alpha_{0};\beta_{0})}\\ &\quad\times \left(\frac{1}{n}\sum_{i=1}^{n} \frac{\partial r}{\partial \eta_{i\alpha}}(\eta_{i\alpha})\frac{\partial \eta_{i\alpha}}{\partial{\alpha} } \frac{\partial \eta_{i\alpha}}{\partial{\alpha^{\prime}}}\frac{\partial r}{\partial \eta_{i\alpha}}(\eta_{i\alpha}) \right)_{\alpha_0}^{-1} \left(\frac{1}{\sqrt{n}}\sum_{i=1}^{n} \frac{\partial r}{\partial \eta_{i\alpha}}(\eta_{i\alpha})\frac{\partial \eta_{i\alpha}}{\partial{\alpha}} \left(T_{i}-r(\eta_{i\alpha})\right)\right)_{\alpha_0} \end{aligned} $$

with $\eta _{i\alpha } = w_{i}^{\prime }\alpha $. The latter is derived from the asymptotic approximation of $\sqrt {n}(\hat {\alpha }-\alpha _{0})$ using a similar Taylor-Lagrange expansion for the first-stage nonlinear least squares regression. The previous expansion leads to the covariance matrix of $\hat {\beta }$ of the form

10 $${} \begin{aligned} {\mathbb{V}ar}(\hat{\beta}) &= \frac{1}{n} \left(A_{22}^{-1} S_{2} A_{22}^{-1^{\prime}} \,+\, A_{22}^{-1}A_{21} A_{11}^{-1} S_{1}A_{11}^{-1^{\prime}}A_{21}^{\prime} A_{22}^{-1^{\prime}}\right.\\ &\left.\quad- A_{22}^{-1} S_{21} A_{11}^{-1^{\prime}}A_{21}^{\prime} A_{22}^{-1^{\prime}}\right). \end{aligned}  $$

Under the assumption of independence between observations, the matrices involved in this covariance matrix are given by

$${} \begin{aligned} A_{11} &\,=\, p\lim\! \left(\frac{1}{n} \sum_{i=1}^{n} \frac{\partial r}{\partial \eta_{i\alpha}}(\eta_{i\alpha})\frac{\partial \eta_{i\alpha}}{\partial{\alpha} } \frac{\partial \eta_{i\alpha}}{\partial{\alpha^{\prime}}}\frac{\partial r}{\partial \eta_{i\alpha}}(\eta_{i\alpha}) \right)_{\alpha_{0}}\\ A_{21} &\,=\, p\lim\! \left(\frac{1}{n} \sum_{i=1}^{n} \frac{\partial F_{\alpha}}{\partial \eta_{i\beta}}(\eta_{i\beta})\frac{\partial \eta_{i\beta}}{\partial{\alpha} } \frac{\partial \eta_{i\beta}}{\partial{\beta^{\prime}}}\frac{\partial F_{\alpha}}{\partial \eta_{i\beta}}(\eta_{i\beta}) \right)_{(\alpha_{0};\beta_{0})}\\ A_{22} &\,=\, p\lim\! \left(\frac{1}{n} \sum_{i=1}^{n} \frac{\partial F_{\alpha}}{\partial \eta_{i\beta}}(\eta_{i\beta})\frac{\partial \eta_{i\beta}}{\partial{\beta} } \frac{\partial \eta_{i\beta}}{\partial{\beta^{\prime}}}\frac{\partial F_{\alpha}}{\partial \eta_{i\beta}}(\eta_{i\beta}) \right)_{(\alpha_{0};\beta_{0})}\\ S_{1} &\,=\, p\lim\! \left(\frac{1}{n} \sum_{i=1}^{n} \frac{\partial r}{\partial \eta_{i\alpha}}(\eta_{i\alpha})\frac{\partial \eta_{i\alpha}}{\partial{\alpha} } U_{i\alpha}^{2}\frac{\partial \eta_{i\alpha}}{\partial{\alpha^{\prime}}}\frac{\partial r}{\partial \eta_{i\alpha}}(\eta_{i\alpha}) \right)_{\alpha_0}\\ S_{2} &= p\lim\! \left(\!\frac{1}{n} \sum_{i=1}^{n}\! \frac{\partial F_{\alpha}}{\partial \eta_{i\beta}}(\!\eta_{i\beta})\frac{\partial \eta_{i\beta}}{\partial{\beta} } U_{i\beta}^{2} \frac{\partial \eta_{i\beta}}{\partial{\beta^{\prime}}}\frac{\partial F_{\alpha}}{\partial \eta_{i\beta}}(\!\eta_{i\beta})\!\right)_{\!(\alpha_{0};\beta_{0})}\\ S_{21} &\,=\, p\lim\! \left(\!\frac{1}{n} \!\sum_{i=1}^{n}\! \frac{\partial F_{\alpha}}{\partial \eta_{i\beta}}(\!\eta_{i\beta}\!)\frac{\partial \eta_{i\beta}}{\partial{\beta} } U_{i\beta} U_{i\alpha} \frac{\partial \eta_{i\alpha}}{\partial{\alpha^{\prime}}}\frac{\partial r}{\partial \eta_{i\alpha}}(\!\eta_{i\alpha}\!)\!\!\right)_{\!(\alpha_{0};\beta_{0})} \end{aligned} $$

where $p\lim $ denotes the limite in probability, *U*_*α*_=*T*−*P*_*α*_, *U*_*β*_=*y*−*P*_*β*_, with *P*_*α*_=*r*(*wα*) and *P*_*β*_=*F*_*α*_(*Xβ*).

An estimation of this covariance matrix can be obtained using the plug-in estimator of each matrix involved in its expression. Let $P_{\hat {\alpha }} = r(w\hat {\alpha }), P_{\hat {\beta }} = F_{\hat {\alpha }}(X\hat {\beta }),\ U_{\hat {\alpha }} = T-P_{\hat {\alpha }}$ and $ U_{\hat {\beta }} = y-P_{\hat {\alpha }},$ then the corresponding estimator of matrices at equation (10) are such that

$$\begin{aligned} \hat{A}_{11} &= \frac{1}{n} \left[P_{\hat{\alpha}}(1-P_{\hat{\alpha}})w \right]^{\prime} [P_{\hat{\alpha}}(1-P_{\hat{\alpha}})w],\\ \hat{A}_{21} &= \frac{\hat{\beta}_{u}}{n} \left[P_{\hat{\beta}}^{2}\left(1-P_{\hat{\beta}}\right)^{2}X\right]^{\prime} [P_{\hat{\alpha}}(1-P_{\hat{\alpha}})w]\\ \hat{A}_{22} &= \frac{1}{n} \left[P_{\hat{\beta}}\left(1-P_{\hat{\beta}}\right)X\right]^{\prime} \left[P_{\hat{\beta}}\left(1-P_{\hat{\beta}}\right)X\right],\\ \hat{S}_{1} &= \frac{1}{n} \left[P_{\hat{\alpha}}(1-P_{\hat{\alpha}})U_{\hat{\alpha}}w\right]^{\prime} [P_{\hat{\alpha}}(1-P_{\hat{\alpha}})U_{\hat{\alpha}}w]\\ \hat{S}_{2} &= \frac{1}{n} \left[P_{\hat{\beta}}(1-P_{\hat{\beta}})U_{\hat{\beta}}X\right]^{\prime} \left[P_{\hat{\beta}}\left(1-P_{\hat{\beta}}\right)U_{\hat{\beta}}X\right],\\ \hat{S}_{21} &= \frac{1}{n} \left[P_{\hat{\beta}}\left(1-P_{\hat{\beta}}\right)U_{\hat{\beta}}X\right]^{\prime} [P_{\hat{\alpha}}(1-P_{\hat{\alpha}})U_{\hat{\alpha}}w]. \end{aligned} $$

## Appendix B: Instrument strength and confounding level

We give bellow the computation of instrument strength and confounding level

- **Instrument strength**
  The strength of an instrument results from the correlation between it and the corresponding endogenous variable. In the model considered here, the strength of an instrument *Z* is given by ${\mathbb {C}orr}(T,Z) = \frac {\mathbb {C}ov(T,Z)}{\sqrt {\mathbb {V}ar(T)}\sqrt {\mathbb {V}ar(Z)}}$. As the treatment has a causal link with other covariables *Z*,*X*_1_,*X*_2_ and *X*_*u*_, we have
  $${} \begin{aligned} {\mathbb{V}ar}(T) &= {\mathbb{V}ar}_{Z} [\!{\mathbb{E}}(T|Z,X_{1},X_{2},X_{u})]\\ &\quad+ {\mathbb{E}}_{Z}[{\mathbb{V}ar}(T|Z,X_{1},X_{2},X_{u})]. \end{aligned} $$
  If we consider only the explanatory effect of the instrument in treatment *T* and replace other covariables and the confounder by their average effect, we have ${\mathbb {V}ar}(T) = {\mathbb {V}ar}_{Z} \left (\frac {1}{1+A_Z}\right) + {\mathbb {E}}_{Z}\left (\frac {A_Z}{(1+A_{Z})^{2}}\right)$ with *A*_*Z*_=*exp*(−(*α*_0_+*Zα*_*z*_+*μ*_1_*α*_1_+*μ*_2_*α*_2_+*μ*_*u*_)),$\mu _{1} = {\mathbb {E}}(X_{1}), \mu _{2} = {\mathbb {E}}(X_{2})$ and $\mu _{u} = {\mathbb {E}}(X_{u})$. This variance may then be written
  11 $$\begin{array}{*{20}l} {}{\mathbb{V}ar}(T) &=& {\mathbb{E}}_{Z} \left(\!\frac{1}{1+A_Z}\!\right)^{2}\,-\, \left(\!{\mathbb{E}}_{Z} \left(\!\frac{1}{1+A_Z}\!\right)\!\right)^{2}\\ &&+ {\mathbb{E}}_{Z}\!\left(\!\frac{A_Z}{(1+A_{Z})^{2}}\!\right) \end{array} $$
  12 $$\begin{array}{*{20}l} &=& {\mathbb{E}}_{Z} \!\left(\!\frac{1+A_Z}{(1+A_{Z})^{2}}\!\right)\,-\, \left(\!{\mathbb{E}}_{Z}\! \left(\!\frac{1}{1+A_Z}\!\right)\!\right)^{2} \end{array} $$
  13 $$\begin{array}{*{20}l} &=& {\mathbb{E}}_{Z}\! \left(\!\frac{1}{1+A_Z}\!\right)\! -\! \left(\!{\mathbb{E}}_{Z}\! \left(\!\frac{1}{1+A_Z}\!\right)\!\right)^{2}. \end{array} $$
  Considering a dichotomous instrument *Z* having the Bernoulli distribution *B*(*p*), we have ${\mathbb {E}}_{Z}\left (\frac {1}{1+A_Z}\right) = \frac {p}{1+A_1} + \frac {1-p}{1+A_0},$ where *A*_*j*_,*j*=0,1 is *A*_*Z*_ with *Z* replaced by *j*. We finally obtain
  $${} \begin{aligned} {\mathbb{V}ar}(T) = \left(\!\frac{p}{1+A_1} + \frac{1-p}{1+A_0}\!\right)\!\left(\!1\,-\,\left(\!\frac{p}{1+A_1} + \frac{1-p}{1+A_0}\right)\!\right)\!. \end{aligned} $$
  We also have
  14 $$\begin{array}{*{20}l} {\mathbb{E}}(T) &=& {\mathbb{E}}_{Z}({\mathbb{E}}(T|Z,X_{1},X_{2},X_{u})) \end{array} $$
  15 $$\begin{array}{*{20}l} &=& {\mathbb{E}}_{Z}\left(\frac{1}{1+A_Z}\right) \end{array} $$
  16 $$\begin{array}{*{20}l} &=& \frac{p}{1+A_1} + \frac{1-p}{1+A_0} \end{array} $$
  and then ${\mathbb {E}}(Z){\mathbb {E}}(T) = \frac {p^{2}}{1+A_1} + \frac {p(1-p)}{1+A_0}$. Furthermore, ${\mathbb {E}}(ZT) = {\mathbb {E}}_{Z}({\mathbb {E}}(ZT|Z,X_{1},X_{2},X_{u})) = {\mathbb {E}}_{Z}(Z{\mathbb {E}}(T|Z,X_{1},X_{2},X_{u}))$ which leads to ${\mathbb {E}}(ZT) ={\mathbb {E}}_{Z}\left (\frac {Z}{1+A_Z}\right) = \frac {p}{1+A_1}$. The covariance between *Z* and *T* is then given by
  17 $$\begin{array}{*{20}l} {\mathbb{C}ov}(Z,T) &=& {\mathbb{E}}(ZT) - {\mathbb{E}}(Z){\mathbb{E}}(T) \end{array} $$
  18 $$\begin{array}{*{20}l} &=& \frac{p}{1+A_1} - \frac{p^{2}}{1+A_1} - \frac{p(1-p)}{1+A_0}\end{array} $$
  19 $$\begin{array}{*{20}l} &=& p(1-p)\left(\frac{1}{1+A_1} - \frac{1}{1+A_0}\right). \end{array} $$
  The correlation between *Z* and *T* is
  20 $$ {} {\mathbb{C}orr}(T,Z) = \frac{\left(\frac{1}{1+A_1}-\frac{1}{1+A_0}\right)\sqrt{p(1-p)}}{\sqrt{\left(\frac{p}{1+A_1}+\frac{1-p}{1+A_0}\right)\left(1-\left(\frac{p}{1+A_1}+\frac{1-p}{1+A_0}\right)\right)}}.  $$
  Then for a given instrument *Z* with *p* fixed in (20), *α*_0_, *α*_*z*_,*α*_1_ and *α*_2_ can be chosen to reach a value of *A*_*Z*_ that leads to a desired value of ${\mathbb {C}orr}(T,Z)$.
  Another criterion that could be used to quantify an instrument’s strength is the correlation between *T*^∗^=*α*_0_+*Zα*_*z*_+*X*_1_*α*_1_+*X*_2_*α*_2_+*X*_*u*_ and the instrument *Z*. Since ${\mathbb {C}ov}(Z,T^{*}) ={\mathbb {C}ov}(Z,\alpha _{0} + Z \alpha _{z}+ X_{1}\alpha _{1}+ X_{2}\alpha _{2}+ X_{u}) = \alpha _{z}{\mathbb {V}ar}(Z)$ and $ {\mathbb {V}ar}(T^{*}) = \alpha _{z}^{2}{\mathbb {V}ar}(Z) + \alpha _{1}^{2}{\mathbb {V}ar}(X_{1}) + \alpha _{2}^{2}{\mathbb {V}ar}(X_{2}) + {\mathbb {V}ar}(X_{u}),$ under the assumptions on these variables, we have
  21 $$ {\mathbb{C}orr}(Z,T^{*}) = \frac{\alpha_{z}\sqrt{p(1-p)}}{\left(\alpha_{z}^{2}p(1-p) + \alpha_{1}^{2} \sigma_{1}^{2} +\alpha_{2}^{2} \sigma_{2}^{2} + \sigma_{u}^{2} \right)^{1/2}}  $$
  where $\sigma _{i}^{2} ={\mathbb {V}ar}(X_{i}) $ and $\sigma _{u}^{2} = {\mathbb {V}ar}(X_{u})$.
- **Confounding level**
  A straightforward calculation as above leads to the following correlation between *T*^∗^ and *X*_*u*_ that expresses the level of confounding.
  22 $${} {\mathbb{C}orr}(X_{u},T^{*}) = \frac{\sigma_u}{\left(\alpha_{z}^{2}p(1-p) + \alpha_{1}^{2} \sigma_{1}^{2} +\alpha_{2}^{2} \sigma_{2}^{2} + \sigma_{u}^{2} \right)^{1/2}}.  $$

## Appendix C: F-statistics for each scenario

The following tables display the equivalent of the first-stage F-statistics in linear regression (see [33]) testing instrument exclusion in the treatment choice model.

**Table 3:** Monte-carlo mean of F-statistics in each scenario using the proportion of patients who received the same treatment as proxy of instrument

*n*	Level	Weak	Mod	Strong
30000				
	High	14.14	101.32	432.00
	Med	16.95	105.93	458.32
	Low	17.65	108.05	465.88
20000				
	High	11.47	69.15	290.29
	Med	13.49	71.53	302.10
	Low	15.93	75.59	315.68
10000				
	High	8.96	36.64	147.29
	Med	11.76	39.26	152.53
	Low	12.48	42.80	161.04

Legend: Low, Med (Medium), High denote level of confounding whereas Weak, Mod (Moderate), Strong stand for instrument strength. The number *n* with values 10000, 20000 and 30000 stands for the sample size

**Table 4:** Monte-carlo mean of F-statistics in each scenario using the treatment prescribed to the last patient as proxy of instrument

*n*	Level	Weak	Mod	Strong
30000				
	High	8.13	13.99	45.30
	Med	12.18	15.66	49.45
	Low	13.50	16.18	50.83
20000				
	High	8.47	10.94	32.26
	Med	11.98	12.11	32.99
	Low	13.35	13.79	31.93
10000				
	High	8.25	8.07	18.48
	Med	10.69	9.80	18.88
	Low	14.17	10.38	19.76

Legend: Low, Med (Medium), High denote level of confounding whereas Weak, Mod (Moderate), Strong stand for instrument strength. The number *n* with values 10000, 20000 and 30000 stands for the sample size

**Table 5:** Performances of methods using instrument *pr*

		Instrument strength											
		Weak				Mod				Strong			
Level	Method	*rB*	*sd*	*rMSE*	*pval*	*rB*	*sd*	*rMSE*	*pval*	*rB*	*sd*	*rMSE*	*pval*
High	Tr	1.62	0.23	0.24	0.06	0.52	0.26	0.24	0.04	1.93	0.40	0.32	0.06
	Conv	46.14	0.23	1.40	1.00	42.40	0.26	1.29	1.00	39.62	0.39	1.23	1.00
	2SRI	14.98	0.66	0.85	0.13	9.41	0.47	0.53	0.09	8.49	0.44	0.53	0.10
	GMM	72.01	315.65	5.06	0.27	75.50	109.91	4.70	0.32	71.32	60.00	4.25	0.40
Med	Tr	0.58	0.25	0.23	0.05	1.25	0.31	0.30	0.07	1.61	0.48	0.32	0.04
	Conv	25.40	0.25	0.80	0.96	23.98	0.31	0.78	0.85	21.37	0.48	0.72	0.63
	2SRI	9.93	0.66	0.70	0.07	6.90	0.54	0.61	0.08	5.45	0.54	0.54	0.09
	GMM	36.76	47.90	43.89	0.40	32.85	32.04	2.17	0.51	44.82	41.87	3.36	0.35
Low	Tr	0.66	0.26	0.26	0.07	1.51	0.33	0.28	0.04	2.20	0.55	0.35	0.05
	Conv	7.86	0.26	0.35	0.15	7.96	0.33	0.37	0.11	7.72	0.55	0.41	0.10
	2SRI	6.43	0.71	0.74	0.07	5.76	0.64	0.62	0.06	5.91	0.66	0.67	0.10
	GMM	22.04	31.69	2.42	0.49	26.15	31.68	3.49	0.64	43.12	25.42	4.14	0.46

Legend: Tr = True model, Conv = Conventional model, 2SRI = Two-Stage Residual Inclusion, GMM = Generalized Method of Moment. Low, Med (Medium), High denote the level of confounding whereas Weak, Mod (Moderate), Strong stand for instrument strength. For the criteria, *rB*= relative bias (*%*),*sd*= standard deviation, *rMSE*= root Mean Squares error and *pval*= non-coverage probabilities

## Appendix D: Description of simulation model and parameters values, results of the second scenario

For all scenarios, the model generating the binary outcome is the index function

$$Y_{i} = 1(Y_{i}^{*}-\varepsilon_{i}>0), $$

with $Y_{i}^{*} = \beta _{0} + T_{i}\beta _{t}+ X_{1i}\beta _{1}+ X_{2i}\beta _{2}+ X_{ui}\beta _{u} $ and *ε*_*i*_ the standart logistic distribution. *T*_*i*_ is the observed binary treatment of the individual *i*,*X*_1*i*_ and *X*_2*i*_ some characteristics of patient *i* and *X*_*ui*_ the unmeasured confounding factor. Besides *β*_0_ the intercept, *β*_0_,*β*_*t*_,*β*_1_,*β*_2_ and *β*_*u*_ are parameters related to *T*, *X*_1_, *X*_2_ and *X*_*u*_ respectively. We fixed these parameters *β*_0_=−0.6, *β*_*t*_=3, *β*_1_=1, *β*_2_=1, and *β*_*u*_=1 to keep the prevalence of event less than 5*%*.

**Table 6:** Number of samples among 500 leading to outliers in GMM estimation

*n*	Level	Weak	Mod	Strong
	High	241	208	155
	Med	220	188	185
	Low	124	127	145

Legend: Low, Med (Medium), High denote level of confounding whereas Weak, Mod (Moderate), Strong stand for instrument strength

The treatment choice for the *i*^*th*^ patient was generated from a Bernoulli model with success probability *p*_*i*_ which depends on the patient’s characteristics *X*_1_∼*N*(−2,1) and *X*_2_∼*N*(−3,1), on a binary instrument *Z*∼*b*(0.7) and on the confounding factor *X*_*u*_∼*N*(0,*σ*_*u*_). The probability *p*_*i*_ is given by *p*_*i*_=*F*(*α*_0_+*Z*_*i*_*α*_*z*_+*X*_1*i*_*α*_1_+*X*_2*i*_*α*_2_+*X*_*ui*_), where *F* denotes the logistic distribution function. The standard deviation *σ*_*u*_ of the confounding factor takes values 0.5, 1 and 1.5 corresponding to Low, Medium and high level of confounding respectively. For a fixed level of confounding, we varied only *α*_*z*_ value over {1,2,3} of which each element corresponds to an instrument strength: 1 for “Low” instrument, 2 for “Moderate” and 3 for “High” instrument. All other parameters in the treatment choice model remain constant (*α*_0_=0.2, *α*_1_=2 and *α*_2_=1.2). The data are generated using the R function sim2Logit2().

We investigated the performances of methods in the context of rare exposures (2 to 6*%*), and rare events (less than 5*%*). To check whether the results obtained remain valid in the context of higher exposure (near 50*%*), we design new simulations in which only the intercepts *α*_0_ and *β*_0_ are modified in the previous design. We held all other parameters constant and fixed *α*_0_=5 and *β*_0_ = −2.3. The prevalence of exposure ranged then between 26*%* and 45*%* whereas that of event is maintained lower than 6*%*. We present in Table 5 the results from this second scope over 500 Monte Carlo samples of size 30000. Table 6 displays the number of Monte Carlo samples with outliers.

This function allows to simulate a compound logistic model with covariates


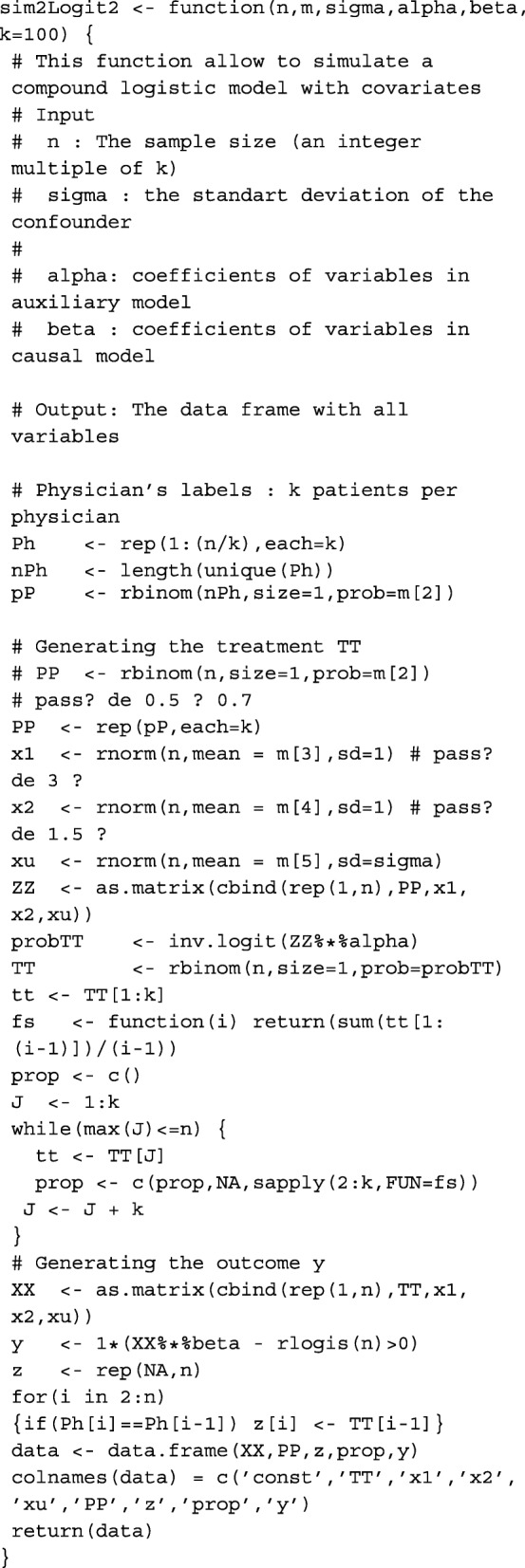


## Abbreviations

2SLS: Two-Stage Least Squares
2SPS: Two-Stage Predictor Substitution
2SRI: Two-Stage Residual Inclusion
GMM: Generalized Method of Moment
Inserm: Institut national de la santé et de la recherche médicale
IV: Instrumental variable
OR: Odd Ratio
PP: Physician Preference
pval: non coverage probability
rB: Relative Bias
rMSE: root Mean Square Error
sd: standard deviation
UVSQ: Université de Versailles Saint-Quentin-en-Yvelines
